# Current and Potential Applications of Artificial Intelligence in Gastrointestinal Stromal Tumor Imaging

**DOI:** 10.1155/2020/6058159

**Published:** 2020-11-26

**Authors:** Cai-Wei Yang, Xi-Jiao Liu, Si-Yun Liu, Shang Wan, Zheng Ye, Bin Song

**Affiliations:** ^**1**^ Department of Radiology, West China Hospital, Sichuan University, Chengdu 610041, Sichuan Province, China; ^2^GE Healthcare (China), Beijing 100176, China

## Abstract

The most common mesenchymal tumors are gastrointestinal stromal tumors (GISTs), which have malignant potential and can occur anywhere along the gastrointestinal system. Imaging methods are important and indispensable of GISTs in diagnosis, risk staging, therapy, and follow-up. The recommended imaging method for staging and follow-up is computed tomography (CT) according to current guidelines. Artificial intelligence (AI) applies and elaborates theses, procedures, modes, and utilization systems for simulating, enlarging, and stretching the intellectual capacity of humans. Recently, researchers have done a few studies to explore AI applications in GIST imaging. This article reviews the present AI studies in GISTs imaging, including preoperative diagnosis, risk stratification and prediction of prognosis, gene mutation, and targeted therapy response.

## 1. Introduction

The most frequent mesenchymal tumors from the gastrointestinal system are gastrointestinal stromal tumors (GISTs), with a prevalence of 14–20 cases per million [1]. GISTs can occur anywhere in the gastrointestinal system, with 50–60% located in the stomach, 30–35% sited in the small intestine, 5% originated in the colon and rectum, and less than 1% in the esophagus [2]. The surgical operation is the first treatment way for GISTs with malignant potential.

Imaging methods are important and indispensable of GISTs in diagnosis, staging, follow-up, and surveilling adjuvant therapy response [3]. The recommended imaging method for GISTs classification is computed tomography (CT) according to current guidelines [4], while magnetic resonance imaging (MRI) or enhanced endoscopic ultrasonography (EUS) could be replacements for iodine allergic or pregnant patients, [18F]-fluorodeoxyglucose positron emission tomography (PET)–CT can be conducive for early phase monitoring of tumor response to tyrosine kinase inhibitor (TKI) therapy [4].

At present, the clinical images practice mainly depends on the subjective interpretation by radiologists of morphological signs such as the location, margin, contour, size, attenuation, growth type, and enhancement degree. With the application and popularization of high-end multislice spiral CT, high-quality images containing rich digital information are available and prevalent, promoting artificial intelligence (AI) techniques to mine and process the big data deep in the images. Recently, an explosion of AI research emerged, particularly in the medicine field.

Recently, researchers have reported a few studies exploring the AI applications in GISTs imaging, including preoperative diagnosis, risk stratification and prediction of prognosis, gene mutation, and targeted therapy response. The current article aims to review the AI imaging studies in GISTs in relation to these four aspects.

## 2. Artificial Intelligence

As an information science, AI applies and elaborates theses, procedures, modes, and utilization systems for simulating, enlarging, and stretching intellectual capacity of humans [5].

Lambin et al. [6] initially proposed the notion of radiomics in 2012, which consisted of a computer-aided operating instrument derived from a great number of features from radiographic images. This technique as a new imaging technology, which can provide objective image information that cannot be recognized by the naked eye, is more detailed than the personal image interpretation by radiologists' vision. And texture analysis can quantitatively evaluate and extract the characteristics of tumors and can assess tumor heterogeneousness related to histopathological components in tumor tissues and mainly influenced by tumor neovascularization (vascularization formation and vascular permeability), tumor cellular structure, tumor cell density, and microcirculation deformation. Such quantitative-feature-based method could be of clinical associations of tumor diagnosis, staging, prognosis, and therapy.

In detail, radiomic texture is mainly composed of statistical texture, morphology-based texture, and transform-based texture. The statistical texture is formed on assessing texture as a measurement of the gray levels statistical properties based on processing the region of interest (ROI). It mainly includes (1) first-order statistical features, depicting distributed pixels in an image, such as histogram analysis; (2) second-order statistical features, as well as texture features, represent spatial relations between pixels and corresponding pair ratios, including gray-level cooccurrence matrix (GLCM), gray-level difference matrix (GLDM), gray-level run-length (GLRLM), gray-level size zone matrix (GLSZM), and neighborhood gray-tone difference matrix (NGTDM); (3) higher-order statistical features refer to the features extracted after applying filters or mathematical transformations for images, such as first-order and textural features extracted from the wavelet-filtered, Laplacian of Gaussian (LoG)-filtered, or local binary pattern (LBP) filtered images. The transform-based analysis includes texture characteristic extraction based on wave spectral statistical properties of and characterization of the global periodicity of gray level by high-energy apices and their varied types in the spectrum.

**Figure 1:**
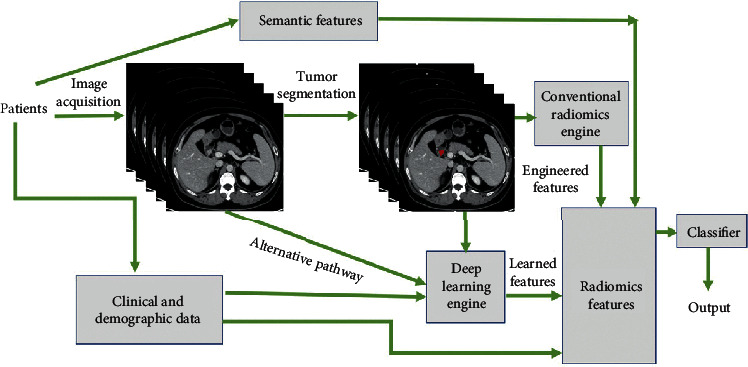
A representative radiomics workflow is composed of four tasks: image acquisition, tumor segmentation, features extraction, and subsequent statistical analysis.The patient in Figure 1 had a small gastrointestinal stromal tumor in the duodenum.

Morphology-basing method incorporates the decomposition of an image into basic units and the determination of the rules required to assemble a given image based on these basic units. All of the above methods consist in various descriptors. The detailed descriptions are presented in Table 1. In a simplistic way, a representative radiomics workflow is composed of four tasks: image attainment, image segmentation, parameter extraction, and statistical analysis (Figure 1).

Radiogenomics, as well as an encouraging novel exemplification, has the potential to extend and expand traditional radiographic images into the field of molecular and genomic imaging [7]. It aims to correlate image features with patterns of gene expressions, gene mutations, and any other genes associated traits, promoting a deeper level explanation of tumor heterogeneity and the development of imaging biomarkers [8].

Deep learning is a group of machine learning algorithms extracting deep features of the input image via multiple hidden layers [9]. Such multilayered computational models can progressively learn representations of data during multilevel abstraction [10]. A neural network is an embranchment of machine learning that organizes the basic structure of a deep learning network [11]. The models of deep learning algorithms used in medical imaging processing include Sparse Autoencoder, Convolutional Neural Network (CNN), Deep Belief Network, Restricted Boltzmann Machine, and Residual Neural Network (ResNet) [10]. Among various deep learning networks, CNN is the most popular architecture, and a further improved neural network included more computational layers.

## 3. Diagnosis and Differential Diagnosis

GISTs represent a distinct histopathological group of subepithelial tumors. A broad range of other mesenchymal tumors can also manifest similar imaging features with GISTs, while the two groups have distinguished prognosis and treatment. Previous studies have differentiated GISTs from other mesenchymal tumors based on tumor location, margin, contour, size, attenuation, growth type, enhancement degree, and necrosis [12–17]. However, it is still difficult to discriminate GISTs with a diameter less than 5 cm from other mesenchymal tumors, only counting on subjective imaging interpretations. There is a vacancy of AI research in this area, we look forward to more AI researches to dig new data in this field.

Clinically, the preoperative diagnosis of GISTs around the periampullary area poses a dilemma in conventional imaging performance. Rather, pancreatic ductal adenocarcinomas (PDACs), duodenal adenocarcinomas (DACs), and GISTs differed in surgery procedures and prognosis [15–17]. Recently, Lu et al. [18] retrospectively studied 74 patients with duodenal tumors around the periampullary area: 26 DACs, 20 DACs, and 28 GISTs. Volumetric histogram analysis was performed on enhanced multidetector CT images based on tumor heterogeneity. They concluded that some parameters of CT histogram analysis of periampullary tumors could be valuable for diagnostic differentiating DACs, PDACs, and GISTs arising from the periampullary area. However, the sample size and tumors type involved in this article are limited. Further researches with more sample capacity and various kinds of tumors will reinforce AI application in GISTs diagnosis.

## 4. Prediction of Risk Stratification and Prognosis

Several risk assessment systems for postoperative recurrence of GISTs have been proposed and evolved over the years, including the National Institute of Health (NIH) criteria, Armed Forces Institute of Pathology (AFIP) standard, and National Comprehensive Cancer Network (NCCN) risk classification. In 2008, modifications of the NIH criteria were proposed, which incorporated tumor location, size, mitotic count, and tumor rupture. The criteria of the recurrence risk categorized into four groups (including very-low-risk group, low-risk group, intermediate-risk group, and high-risk group) and is accepted worldwide [19]. Imaging can provide more findings related to the risk stratification of GISTs. According to previous studies [20–24], tumor growth mode is related to the risk, and the risk level of GISTs with exophytic or mixed growth mode is high. It has also been suggested that the enhancement type, boundary, enlarged blood vessels, necrosis, calcification, and invasions to adjacent organs are connected to the tumor risk stratification.

The differences among the observers of subjective evaluations urged researchers to find more stable and more objective parameters and indicators. The texture analysis could extract more information hidden from medical images, which cannot be identified by subjective visual interpretation. In theory, the judgment efficiency of texture analysis of GISTs risk stratification is better than the conventional imaging [24, 25]. Nine studies have researched the performance of CT-derived radiomic signature for risk stratification [24–32], and one study evaluated EUS-derived texture [33] associated with risk stratification. The details are summarized in Table 2. In CT-derived analysis, four studies have applied NIH criterion or modified NIH criterion for GISTs malignant risk classification [25–28], while three studies were determined on NCCN guideline [24, 29, 30] and one study without clear guideline [33] and one study used Ki-67 expression standard [32]. Two of the four NIH studies based on NIH risk classification only evaluated CT textural parameters [26, 27]. The remaining two studies combined and compared conventional visual CT findings and clinical indexes models [25, 28].

In 2018, Feng et al. [26] retrospectively reviewed 90 intestinal GISTs patients. GISTs risk levels were evaluated by CT-derived histogram features that were compared according to modified NIH risk classification. They believe that volumetric CT texture features show the feasibility to be biomarkers for distinguishing low-risk, intermediate-risk, and high-risk intestinal GISTs (area under the curve (AUC) = 0.830, *P* < 0.001). However, some studies have reported contradictory results with the present study [29, 34–36]. We speculate that the differences in ROI delineation methods, and differences between enhanced and unenhanced CT-derived texture features might be associated. In this study, the numbers of some risk groups of intestinal GISTs were limited and the author combined some groups. Moreover, this study only applied first-order statistical radiomics features. It will require further studies to explain the controversy.

Another research [27] constructed a radiomics model using multiple-order statistical radiomics features based on contrast-enhanced CT to noninvasively predict malignant-transformation potential and mitotic indexes of GISTs. In this research, the patients were classified as low- (including very-low-risk GISTs, low-risk GISTs, and intermediate-risk GISTs) and high-malignant-transformation-potential group (high-risk GISTs) based on the NIH criterion, and the sample size is enlarged with 333 numbers in total (training cohort = 233 and validation cohort = 100). The radiomics model showed a good predictive performance in differentiating high-from low-malignant-transformation-potential GISTs with an AUC value of 0.882 in the training group and 0.920 in the validation group.

The above two studies have only constructed radiomics model, and a single radiomics model could not utilize and compare the performance of conventional image findings and clinical information in GISTs' risk stratification. The next two studies [25, 28] compared the accuracy of CT-derived textural parameters, subjective CT parameters, and clinical index models in predicting risk stratification. Yan et al. [28] included 213 intestinal GISTs patients to assess the predictive effect of clinical and subjective imaging findings and multidetector CT texture findings on preoperative risk stratification. They reported that an AUC of the model combining clinical and conventional imaging findings and multidetector CT texture features was 0.943. They deduced that CT texture may be a useful integrated tool for preoperative risk stratification of intestinal GISTs. In 2019, Chen et al. [25] constructed a radiomics nomogram for predicting GISTs malignancy potential. In comparison to conventional CT parameters and clinical indexes, the radiomics model could discriminate low-from high-malignant-transformation-potential group GISTs with a higher AUC value of 0.858. Besides, the generated radiomic nomogram model achieved the highest diagnostic performance, which showed an AUC of 0.867 and 0.847 in the internal and external cohort.

The same predicament for the only usage of radiomics model and limited sample size applied to these studies using NCCN guidelines [29, 30]. Liu et al. [29] found meaningful texture parameters from various phases in differentiating malignancy risks GISTs based on NCCN risk stratification, which was consistent with a previous study [25]. But the sample size is small as no more than 100 patients, and this study only applied first-order statistical radiomics features. With a larger included sample size (total number = 140, training cohort = 100 and validation cohort = 40) and various statistical radiomics features, Zhang et al. [30] highlighted discriminative performance with an AUC value of 0.935 and an accuracy value of 90.2% in the validation set for advanced from nonadvanced GISTs. Further, the radiomics indicated satisfied discriminative performance for four groups of GISTs risk stratification with an AUC value of 0.809 and an accuracy value of 67.5% in the validation set. Nevertheless, these studies did not conduct a direct or indirect correlation among radiomics features, subjective imaging findings, and pathological results.

So then, Choi et al. [24] evaluated and compared the diagnostic performance of CT radiomics parameters and visual CT inspection to predict malignancy grade and mitosis index of GISTs. They found the diagnostic accuracy of special radiomics features was better than visual inspection.

However, the previous studies independently used radiomics methods for pattern classification, without regard to relatively global artificially predefined parameters. Researchers also start to explore the GISTs classification efficiency of deep features obtained by deep learning networks. In 2019, Ning et al. [31] introduced an integrated structure including various features applied to a radiomics model and deep convolutional models and incorporated these features to engage in GISTs categorization. The hybrid structure with the combination of radiomics and CNNs features exhibited better performance with an AUC of 0.882 than that of the conventional CT features model (AUC = 0.774), radiomics model (global features) (AUC = 0.807), and CNN model (local features) (AUC = 0.826). As far as we can tell, this is the initial and exclusive study to apply radiomics model and CNNs for GISTs risk stratification, in which the radiomics parameters are derived from a three-dimensional universal section and deep convolutional features derived from a regional section were combined. This integrated structure enhances not merely model robustness but classifier efficiency as well.

In addition, the risk-related molecules were also predicted by using radiomics methods. The ki-67 index is an important marker related to cell proliferation and tumor heterogeneity [37]. Ki-67 is signified in the majority of the reproducing cells in high level expression, besides G0 cells, and Ki-67 is deemed as a global risk marker of malignant potential in GISTs [38]. Previous literature has also demonstrated that expression of high level Ki-67 indexes is an unrelated risk marker for high-malignancy GISTs [39–41]. A multicenter study [32] has also demonstrated a nomogram that consisted of CT-based radiomics features combined with tumor size indicated significant performance in predicting Ki-67 indexes expression in GISTs, with respective AUCs of 0.801, 0.828, and 0.784 in the training, internal validation, and external validation cohort, respectively. This proved that the Ki-67 indexes expression rate in GISTs was potentially connected with the CT textural signature.

Radiomics methods extended its applicability to various imaging modalities. For EUS-based radiomics, Li et al. [33] performed a EUS-derived radiomics model to differentiate GISTs of the higher-risk classification (intermediate-risk and high-risk) from the lower-risk classification (very-low-risk and low-risk). This model can promote the preoperative diagnosis and supply a beneficial reference for clinicians.

All of the above results show that radiomics is superior to traditional imaging description in predicting the risk stratification of GISTs, which built a foundation for the application of radiomics in the future. However, the existing studies remained insufficient. Present studies only evaluated CT-derived texture. The MRI-derived texture analysis may be more potential to dig hidden information, and quantitative imaging modalities may be useful in precise medical improvement. It should also be noted that at present, the sample sizes of most studies were limited. The inconsistency of scanning parameters, scanners, image acquisition protocol, lesion segmentation, the delineation of ROI, and statistical modeling is also presented. Selection bias of texture parameters extraction also manifested in the statistics of the levy, which leads to the consequence that duplication of research results be questioned. In addition, the conclusions of small samples also brought about poor generalization ability in specific clinical applications. Furthermore, CNN based on deep learning may substantially supplement and extend the applicability of radiomics, in the aspects of feature library or the prediction accuracy, but its effectiveness still remains to be verified.

## 5. Prediction of Gene Mutation

GISTs grow up in the interstitial Cajal cells from the gastrointestinal system [42], and 90% express CD117 antigen (C-KIT) [43], a tyrosinase kinase growth factor receptor [44]. GISTs with KIT exon 11 mutated genes are more responsive to imatinib therapy [45–47], while other molecular mutations respond more poorly to imatinib. In addition, GISTs with KIT exon 9 mutations are more responsive to sunitinib. The connection between CT findings and GISTs genotype has been investigated [48]. GISTs with KIT exon 9 mutation classification have significant linkages with tumor size more than 10 cm, a stronger enhancement grade and greater area of tumor necrosis when compared to those of the KIT exon 11 mutation classification (*P* < 0.05).

In 2018, Xu et al. [49] reported a radiogenomic study on GISTs. They included enhanced CT images of 86 GISTs and performed texture analysis. They found that texture analysis could be of use to discriminate GISTs without KIT exon 11 mutated gene group from those with KIT exon 11 mutated gene group. In addition, the nongastric orientation, lower CD34 staining, and higher radiogenomic signature values were connected with GISTs without the KIT exon 11 mutated gene, which achieved satisfactory diagnostic efficiency in the validation group (AUC = 0.904 − 0.962). However, the sample size of the training cohort and validation cohort was 69 and 17 cases, respectively, and there were only four cases of tumors without KIT 11 exon mutation in the validation cohort, which may have affected the accuracy of the results. Hence, a study with a large number of patients is required to validate these conclusions.

## 6. Response Evaluation of Targeted Therapy

Adjuvant TKIs therapy is suggested for patients with a high recurrence risk of GISTs, and enhanced CT is the recommend imaging method for evaluating treatment response.

The study in [50] constructed and confirmed a predictive nomogram for recurrence-free survival (RFS) of GISTs after surgery without aid treatment based on deep learning (ResNet model). The ResNet nomogram was investigated on enhanced CT and clinicopathological factors including mitotic index of tumor, tumor location, and size. Both the ResNet nomogram and model manifested significant prognostic capabilities in 3- and 5-year RFS in receiver operating characteristic curves. They suggested that ResNet nomogram was supreme to the existing risk stratification standards and clinicopathological nomogram majority of the probability of exceeding reasonable threshold probabilities.

For metastatic GISTs undergoing TKI therapy, Ekert et al. [51] identified 25 GISTs patients with KIT and PDGFR mutations. All patients underwent first-line imatinib therapy and different TKI therapies after disease progression. CT texture features were extracted and associated with response categories according to the modified Choi criterion. They came to the conclusion that some of the CT texture features (GLCM inverse difference, GLCM inverse difference normalized, GLRLM, and NGTDM) correlated with prognosis, progressive-free survival, gene mutations, and treatment regimens.

## 7. Conclusions

Previous studies had some limitations. First, all of the above studies were retrospective. Most of them were the single center and the sample sizes were limited. The restricted number of samples not only limited the setting of imaging radiomics threshold standard, but also imposed restrictions on the training of the models [52]. Second, several image acquisition scanners and parameters were used in the same study, which might reduce the reliability and reproducibility of potential findings. Third, all the studies evaluated CT-derived texture. MRI-derived texture analysis might have more potential to uncover hidden information, and quantitative imaging modalities may be useful for improving precision medicine. In the end, most of the significant texture semantics are statistical terms, which lacked explainable correlations to the specific clinicopathological significance and biological characteristics directly and limited the interpretation of AI in repeatable research and clinical application.

The present studies demonstrated that AI methods including radiomics or deep learning have clinical value for GISTs and built a foundation for future application. Considering the limitations, prospective multicenter studies with large samples are needed. Besides, further standardization of inspection techniques and in-depth excavation of detailed signs will deepen our understanding of GIST imaging. The development of AI imaging in PET-CT and MRI will broaden our exploration. In the future, more AI studies and applications are expected in preoperative prediction of various gene mutations and evaluation of the efficacy of targeted therapies to make continuous progress towards the goal of individualized and accurate treatment.

## Figures and Tables

**Table 1:** Feature metrics extracted in the radiomic analysis of images.

Texture metric	Method (s)	Descriptors
First-order (statistical)	Histogram analysis	Mean, median, kurtosis, skewness, quartiles, minimum, maximum, energy (uniformity), entropy, standard deviation
Second-order (statistical)	GLCM, GLDM, NGTDM, GLRLM, GLSZM	Homogeneity, contrast, autocorrelation, prominence, maximum probability, difference variance, dissimilarity, inverse difference moment, sum entropy, sum variance, sum average, inertia, coarseness, busyness, complexity, texture strength, short run emphasis, long run emphasis, gray-level nonuniformity, run-length nonuniformity, intensity variability, run-length variability, long-zone emphasis, short-zone emphasis, intensity nonuniformity, intensity, zone percentage, variability, size zone variability
Transform (statistical)	Fourier, wavelets, discrete cosine, Gabor, law, LoG, LBP	Metrics assessing magnitude, phase, direction, edge, noise, and other descriptors
Structural analysis	Fractal analysis	Hurst component, mean fractal dimension, standard deviation, lacunarity

*Note.* GLCM = gray-level cooccurrence matrix, GLDM = gray-level difference matrix, NGTDM = Neighborhood gray-tone difference matrix, GLRLM = gray-level run-length, GLSZM = gray-level size zone matrix, LoG = Laplacian of Gaussian, LBP = local binary pattern.

**Table 2:** Details of 10 articles on artificial intelligence in the prediction of GISTs' risk stratification and prognosis.

Author	Year	Nation	Study design	Sample size	Extracted features of AI	Software
Feng C et al. [26].	2018	China	Retrospective	90	First-order statistics: Mean attenuation; 10th, 25th, 50th, 75th, and 90th percentile attenuation; skewness; kurtosis; entropy	CT kinetics
Wang C et al. [27].	2019	China	Retrospective	333 Training cohort = 233 Validation cohort = 100	First-order (histogram), haralick features, GLCM, GLRLM	AK
Chen T et al. [25].	2019	China	Retrospective	222 Training cohort = 130 Validation cohort = 92	GLV, GLRLM, GLSZM, NGTDM, GLSZM	MATLAB
Yan J et al. [28].	2018	China	Retrospective	213	First-order (histogram) gradient features, GLCM, GLRLM	MaZda
Liu S et al. [29].	2018	China	Retrospective	78	First-order (histogram)	Image analyzer
Zhang L et al. [30].	2020	China	Retrospective	140 Training cohort = 100 Validation cohort = 40	First-order features, shape and size features, second-order features (GLCM, GLRLM, GLSZM) features, and haralick features	AK
Choi I et al. [24].	2019	Korea	Retrospective	145	First-order statistics: Mean SD of mean, entropy, MPP, skewness, and kurtosis. Geometry with Gaussian filtration	MATLAB
Ning Z et al. [31].	2018	China	Retrospective	231 Training cohort = 130 Validation cohort = 101	First-order, second-order (GLCM, GLRLM, GLSZM, and NGTDM) features	MATLAB PYTHON
Zhang Q et al. [32].	2020	China	Retrospective	339 Training cohort = 148 |Internal validation cohort = 41 External validation cohort = 150	First-order statistics, features of shape, second-order features (GLCM, GLRLM, GLSZM)	PYTHON
Li X et al. [33]	2020	China	Retrospective	915 Training cohort = 680 Validation cohort = 54 Testing cohort = 181	First-order (histogram), second-order (GLCM, GLRLM, GLSZM, NGTDM) and wavelet-filtered features	MATLAB

*Note.* GLCM = gray-level cooccurrence matrix, GLRLM = gray-level run-length matrix, GLV = gray-level variance, GLSZM = gray-level size-zone matrix, NGTDM = Neighborhood gray-tone difference matrix.

## Abbreviations

GISTs: Gastrointestinal stromal tumors
CT: Computed tomography
MRI: Magnetic resonance imaging
PET-CT: Positron emission tomography-CT
TKI: Tyrosine kinase inhibitor
EUS: Endoscopic ultrasonography
AI: Artificial intelligence
ROI: Region of interest
GLCM: Gray-level cooccurrence matrix
GLDM: Gray-level difference matrix
NGTDM: Neighborhood gray-tone difference matrix
GLRLM: Gray-level run-length
GLSZM: Gray-level size zone matrix
LoG: Laplacian of Gaussian
LBP: Local binary pattern
CNN: Convolutional neural network
ResNet: Residual neural network
PDAC: Pancreatic ductal adenocarcinoma
DAC: Duodenal adenocarcinoma
NIH: National institute of health
AFIP: Armed forces institute of pathology
NCCN: National comprehensive cancer network
AUC: Area under the curve
RFS: Recurrence-free survival.
